# 

**Published:** 1886-07-20

**Authors:** 


VOL. XVI. JULY 20, 1886. NO. 7.

### THE SOUTHERN MEDICAL RECORD.

LIBRARY INDEXED S.G.O.

EDITORS :

T. S. POWELL, M. D. R. C. WORD, M. D.

COLLABORATORS :

J. McF. GASTON, M. D.,
W. D. BIZZELL, M. D.,
W. P. NICOLSON, M. D.,
E. VAN GOIDTSNOVEN, M. D.,
G. G. ROY, M. D.,
A. G. HOBBS, M. D.,
W. S. ELKIN, M. D.,
W. A. CROW, M. D.

Address R. C. WORD, M.D., Managing Editor, Atlanta, Ga.

Published and Entered as Second-class Matter at ATLANTA, GA.

